# Prism Model: Factors that Influence Teaming in Behavioral Health from the Perspectives of Interprofessional Clinicians

**DOI:** 10.1007/s11414-025-09964-0

**Published:** 2025-09-10

**Authors:** Julie Berrett-Abebe, Jocelyn Novella, Michelle Pagnotta

**Affiliations:** 1 Department of Family Therapy & Social Work, Fairfield University, Fairfield, CT USA; 2 Department of Counselor Education, Fairfield University, Fairfield, CT USA

## Abstract

This qualitative study explores what factors influence teaming in behavioral health settings, from the perspective of behavioral health providers. Twenty-four participants from a range of behavioral health professions engaged in semi-structured interviews. Using a grounded theory approach, data were analyzed, and a “prism” model was developed to capture the complexities of behavioral health providers’ perceptions of factors influencing teaming in various mental health and/or substance use disorder treatment programs. Specific model components included: behavioral health context, individual factors, navigating disciplinary-specific approaches, workplace structures, communication as a “throughline,” and varied perceptions of teaming. The prism model is dynamic, acknowledging the role of the individual in the system while also recognizing that participant perceptions of teaming are shaped by environmental and contextual forces. Each pathway is singular, with a variety of interacting factors. A key finding is that while teaming was viewed positively, there was no shared understanding of what teaming meant or whether it was occurring. The article concludes with implications for behavioral health education and practice, including support for new models of behavioral health care that incentivize teaming, expand community supports and peer workforce, prioritize the goals of recovery and wellness, and provide opportunities for more flexible financing.

## Introduction

The behavioral health workforce in the USA is composed of graduate-prepared and licensed professionals in the fields of psychiatry, advanced practice nursing, psychology, social work, counseling, and marriage and family therapy. Many of these professionals have historically operated as independent therapists, not connected to any specific behavioral health system. Newer to behavioral health teams are individuals in roles that most often do not require graduate degrees and leverage individuals’ lived experiences, such as drug and alcohol counselors, peer counselors, community health workers, and behavioral health technicians.^1^ While behavioral health treatment is provided in various configurations of programs and professionals, there are some universal recommendations for enhancing workforce development as a strategy to improve the quality of care. These recommendations include focusing on meeting the needs of clients rather than the narrower interests of individual professions, expanding the workforce to include peer specialists/supporters, and reducing fragmentation and increasing quality of care through enhanced collaboration and recognition of overlapping scopes of practice amongst behavioral health professionals.^2–4^ Some challenges to reform include the competing interests of various behavioral health professions, siloed graduate education, and the lack of gold standard treatment team configurations.^5,6^

One positive trend in health and behavioral health care reform is momentum towards integrated, team-based care. While integrated care is most often defined as bringing together traditional medical and behavioral health care, integration can also refer to working across levels of services (e.g., inpatient and outpatient), placing clients at the center of care, and sharing responsibility for individual and population-level well-being across disciplines.^7^ Integrated care necessitates working collaboratively on teams. In models of integrated care, teaming has included concepts such as individuals across disciplines coming together in support of a shared service goal, often including shared treatment planning, coordination, and regular communication.^8^

There have been concerted efforts to promote teaming in health care through initiatives such as interprofessional education across health professions as well as movement to value-based payments and accountable care organizations, rather than the traditional fee-for-service payments.^9,10^ The Health Resources and Services Administration (HRSA) has also funded training in integrated care for behavioral health providers as a strategy to better prepare the workforce for integration, and the Centers for Medicare & Medicaid Services (CMS) is accepting applications (Fall 2024) for the Innovation in Behavioral Health Model (IBH), which aims to provide integrated, person-centered care within specialty behavioral health settings.^11,12^ However, up to this point, integration initiatives have received more attention in health versus behavioral health settings. Adding to the complexity, behavioral health organizations are often hiring clinicians interchangeably for the same jobs despite different behavioral health master’s degrees, but there has been limited conversation in the literature about the overlapping and unique professional identities, roles, and scope of practice, particularly inclusive of substance use disorder treatment.^13,14^

Previous research has explored: (1) composition and quality of teaming in health settings, and (2) implementation and outcomes of integrated behavioral health in primary care.^15–19^ Across studies that explore implementation of integrated primary care, factors found to influence successful integration include: organizational culture and leadership, licensure and regulation, team attitudes and beliefs, teaming practices and clear professional roles, flexible scheduling, and community context.^18–21^ However, no similar body of literature exists to describe “teaming” in behavioral health settings.

The scant literature that does exist related to teaming in behavioral health settings focuses primarily on mental health (to the exclusion of substance use disorder treatment) and evaluates the benefits and challenges of teaming to behavioral health programs/outcomes and experiences of teaming from the client perspective.^16,18,22^ No literature was identified that explored teaming from the perspectives of behavioral health providers themselves. Understanding behavioral health providers’ perspectives and how these may differ from academic literature, as well as studies on integrated primary care, is necessary to better align education, practice, and aspirations for best practices in behavioral health-specific settings.^23^

Therefore, this study’s research question is: *According to providers in mental health and/or substance use disorder treatment settings, what factors influence teaming in behavioral health settings?*

## Methods

This study employed a grounded theory approach in order to answer the research question above. Grounded theory allows for the researcher to use analytic tools to understand how the characteristics of the participants’ experiences can be synthesized into abstract concepts. These categories lead to a theory which can explain larger phenomena, such as behavioral health teaming.^24^ This study (protocol 4074) was approved by the Institutional Review Board (IRB) at Fairfield University.

### Researcher reflexivity

The first author, JBA, is a licensed clinical social worker with 12 years of direct experience in interprofessional health settings, holds a Ph.D. in social work, and is the Project Director of a behavioral health workforce development grant. The second author, JN, is a Ph.D.-prepared counselor educator, licensed as a professional counselor, and evaluator on a behavioral health workforce development grant. She spent her clinical career working interprofessionally in a college wellness center. The third author, MP, is a licensed professional counselor with five years of direct clinical experience in a range of clinical settings and is the Project Coordinator of a behavioral health workforce development grant.

### Sample

Participants in this study (*N* = 24) had a master’s degree or higher in a behavioral health field (social work, counseling, marriage and family therapy, psychology, psychiatric nurse practitioners, and/or psychiatry) and were licensed to provide behavioral health care (defined as mental health treatment, substance use disorder treatment, or a combination). Inclusion criteria included: currently working with other behavioral health providers (from at least one other discipline), engaged in direct clinical care, and currently working in the state of CT. Just over half of participants identified as licensed professional counselors or social workers. As part of iterative data collection and analysis, researchers purposefully reached out to recruit a psychiatrist, as the role of psychiatry was a prominent theme in the initial data. Participants represented a wide range of ages and years in the field, although 37.5% of participants had been in the field for 5 years or less. Additionally, 62.5% of participants identified as White and 20.8% identified as Latina. The majority of participants were female (92%) (see Table 1). Participants primarily worked in outpatient clinical settings (non-profit outpatient community health [20.8%] and non-profit outpatient methadone [20.8%]). However, other workplace settings included hospital, clinician-owned LLC, eating disorder residential treatment facility, and local government, among others (see Table 2). It is notable that participants worked in diverse behavioral health settings across levels of care (i.e., outpatient, inpatient, partial hospitalization, residential, carceral, etc.). Participants were employed by 14 different behavioral health programs, with five of those programs employing multiple participants. The number of participants in the same workplace ranged from 2 to 4.

**Table 1:** Socio-demographics (*N* = 24)

Variable	*N*	%
**Age**		
20–29	3	12.5
30–39	8	33
40–49	5	20.8
50–59	7	29.2
60–69	1	4.2
**Gender**		
Female	22	92
Male	2	8.3
**Race/Ethnicity**^a^		
Asian	3	12.5
Black/African American	3	12.5
Latina	5	20.8
White	15	62.5
**Profession**		
Licensed alcohol and drug counseling (LADC)	1	4.2
Licensed professional counselor or licensed professional counselor associate (LPC or LPC-A)	8	33.3
Licensed marriage and family therapist (LMFT)	4	16.7
Licensed clinical social worker or licensed master social worker (LCSW or LMSW)	6	25.0
Advanced practice registered nurse (APRN)	3	12.5
Psychiatrist	1	4.2
Doctor of psychology (PsyD)	1	4.2
**# of Years in profession**		
Less than 1 year	2	8.3
1–5	7	29.2
6–10	3	12.5
11–15	4	16.7
16–20	4	16.7
21–25	3	12.5
25–30	1	4.2

^a^ Race/ethnicity numbers do not equal *N* = 24 because some participants identified as more than one race/ethnicity

**Table 2:** Demographics in workplace (*N* = 24)

Type of clinical program^a^	*N*	%	Avg # of disciplines employed by program
Clinician-owned LLC outpatient behavioral health	4	16.7	6
Hospital–inpatient psychiatric	1	4.2	4
Hospital–outpatient mental health treatment program	2	8.3	4.5
Hospital–psych emergency department	1	4.2	3
Non-profit behavioral health care–methadone clinic^b^	5	20.8	4.8
Non-profit domestic and sexual violence agency	2	8.3	3
Non-profit outpatient community mental health	5	20.8	4.6
Non-profit substance use disorder–outpatient	1	4.2	4
Outpatient mental health–local government	2	8.3	2
Residential eating disorder treatment	1	4.2	5

^a^ There are some sites that consider teams to expand beyond the clinical program; this table represents those employed exclusively by the clinical program

^b^ Three of the providers at the non-profit behavioral health care–methadone clinic worked in a corrections setting

### Data collection

Participants were recruited via email employing purposive and snowball sampling. Individuals in researchers’ professional networks were forwarded a flyer by email. They were asked to forward the flyer to others who could contact the researchers directly if interested in participating in the study. A $50 Amazon gift card was offered for participation, which involved a 30-min semi-structured interview via Zoom. Researchers developed a list of interview questions, including prompts. Examples include: 1. When we talk about teaming, we mean having shared treatment goals and working collaboratively on behalf of client care. Did your graduate education prepare you to work with other behavioral health professionals as a team? Is there anything else that prepared you? 2. Think of your latest interaction with a colleague of a different discipline in relation to a client case. What stands out for you about that interaction? Note: The definition of teaming is based on the SAMHSA-HRSA Center for Integrated Health Solutions (CIHS) key elements for the classification of levels of integration of primary care–behavioral health care collaboration.^8^

### Data analysis

Interviews were recorded and transcribed verbatim. Identifying information was removed from transcripts, and each transcript was assigned a number.

Per the grounded theory process described by Saldaña, researchers began with open coding of all raw data individually (348 codes), then moved to axial coding in order to begin developing categories. To describe the process further, initial independent codes were organized into an Excel chart, which served as a codebook for the constant comparison and concept development necessary in grounded theory.^25^ This codebook also aided in evaluating the experiences of various behavioral health disciplines, by allowing for notation of the discipline of each participant expressing a particular idea. Consensus was reached through discussion and constantly returning to the data. Resulting categories (6) and subcategories (16) are described in Table 3.

**Table 3:** Prism model core categories

Categories	Sub-categories	Illustrative quotes (transcript #)
**Prism**		
Individual factors		
	Personal qualities	“I think there has to be a certain level of trust and understanding of each [provider] as an individual, as opposed to seeing them as their discipline… the approach of risk and protective factors are going to be different, depending on the provider that you are presenting to. So I think some of what is important in that environment is to also know the provider that you’re working with and their style and some of the things that you know that they might ask.” (9)
	Clinical expertise	[As a supervisor assigning cases I consider]: “What is their training?… How comfortable are they in their own skin?” (2)
	Cultural background	[New referrals will] “look at my name [and see it’s from a specific cultural background]… I cannot tell you how many phone calls I get where I’ll pick up the phone and they’ll give me the proper “Hello” in that culture, and I’ll give it back. And then I hear, ‘Oh, thank God!’ And because I know how my parents are, and I’ve worked with so many refugees.” (12)
Behavioral health context		
	Siloed nature of care delivery	“From PHP (partial hospitalization), a lot of times they go to IOP (intensive outpatient) and we’re in the same building. We’re technically the same program. But we’re separate. So that’s a barrier, [we don’t communicate] because they’re also very busy in the IOP, right? Even though they’re on the other side of the building.” (10) *Limited resources in the community: “*It’s not a surprise that beds are just extremely limited at this time… However… we didn’t follow up with the actual community resources that we promise people” (9) *Limited resources in the community:* “I wish there were a way to sort of collect all those resources and make them known to everybody and really accessible… I don’t know what that looks like.” (13)
	Financial and insurance systems	“So any extra teamwork is less contact with patients, which mean less billing.” (24) [Describing an internship in a single-payer health system outside the U.S.]: “It was all just understood that… the payment structure was all under one umbrella, so they wanted to utilize it as well as possible and be as effective as possible.” (9) [Experience submitting billing codes for care coordination or collaboration]: “We have [tried]. And to my knowledge, everything we’ve tried has bounced back and not been covered… if we’re not doing a billable session, it’s not something that’s going to be covered.” (23) “Scheduling also impacts like the ability to collaborate in between clients because you barely have the time to use the restroom. So my understanding is, the schedule is created that way due to productivity… it’s very based… in funders and insurance, and all of that more than it is about client care… I understand there’s money at play. But…it can start to impact client care because you’re just burning clinicians out. And then they leave…” (20)
Navigating discipline-specific approaches		
	Prescribers vs. non-prescribers	“We do have a psychiatrist… we kind of leave her alone a bit more, unless there’s an emergency. But she’s… a very traditional psychiatrist, has her little window with the client. We reach out if there’s an emergency or… some disturbing side effect, or something. But… she oversees all the psychiatric meds.” (21) *Power and hierarchy:* [As a non-prescribing master’s level provider]: “There are many times where I know what the medical issue is, and I can’t say anything. I have to kind of humble myself and say it as… an array of symptoms… And I do feel… [medical professionals] are very comfortable talking about clinical needs and having… psychological assessments… But yeah, there’s not… a vice versa.” (21) *Power and hierarchy:* “[APRN’s and psychiatrists] are ultimately the ones that are going sign off like, are we admitting this person? If we are, is it voluntarily, or is it on a physician’s emergency certificate?… So that’s essentially them.” (9)
	Interchangeability of non-prescribers	“It’s actually pretty much the same if you’re in LADC here, an LPC or LPCA or an LCSW as far as outpatient goes. We’re all outpatient counselors, same title, similar responsibilities. Some are fully licensed people… they may provide supervision. That’s probably the biggest difference that I can think of. Other than that we are pretty consistent across the board.” (5)
Operating within workplace structures		
	Physical space	“I don’t work… on the same floor as psychiatry. So sometimes that can be a barrier too. If we were working on the same floor seeing each other every day, there might be a little bit more [understanding].” (11) “Yes, we’re… 2 feet from each other so it’s super easy. We actually used to share an office. Now we don’t. So that was easier to communicate. You just… look to your left… it’s even easier when you’re in the same office.” (10) “We used to have all our clinicians in what we called a “pod.” They were all in one big room, with desks like all around the sides. And now they have separate offices, so there’s a huge dynamic change, because [before] you could just say, ‘Hey, can I run something by you?’ [Before], you could leave a session, and the look on your face says it all, and another clinician says, ‘Hey, are you okay?’ Versus now you’re in your office, you’re all alone.” (18)
	Configurations of on-site/on-line work	“We’re all in person. We’re not virtual. So it’s very easy to just go and have a conversation with another staff person.” (5) “… [weekly meetings] either were postponed for way too long, or they weren’t happening, and at some point… as much as we want to do it in person,… we have to find a better way so that [the weekly meetings] can be consistent. So this platform (Zoom) is consistent.” (4)
	Scheduling	“On a regular day, I am usually scheduled about 7 or 8 hours on the schedule, and most of that time I’m scheduled back-to-back. So it doesn’t really leave much time for anything. So I think… scheduling also impacts… the ability to collaborate in between clients, because you barely have the time to use the restroom.” (20) “I think I can speak for the APRNs and the clinicians, and say, every clinician would love to sit down and talk to the APRN, even if it were for 5 min about every client. And I think the APRN would love to say, ‘What’s happening in the room?’ So I think time is a barrier, because we simply aren’t blessed with 48 h days yet.” (2)
Communication as a “throughline”		
	Relationship-building	“And I think the accessibility of people is really important, because if I can… have my coffee with you, and… just chat in the lunch room about anything, I’m much more likely to come to you with a question versus if you’re only with clients all the time in your office back-to-back, and then you leave. We don’t have time to collaborate. I don’t feel you’re accessible. I don’t feel you’re on my level.” (18)
	Limitations of written communication	“… when we spoke more in person it was more human. It was more of a connection. You were more likely to talk about the fact that you are selling your daughter’s Girl Scout cookies at the end of a conversation. You’re not going to put that in an email. But if you’re chatting by the water cooler you may… I think [being in person] helps the relationships so much and helps the communication be interpersonal. Talking, actually seeing someone, versus like shooting off you know, 3 emails. And that’s it.” (18)
	Scheduled meetings	“So we do have huddles every single day, for, like I said, it’s like a 15 min meeting every day… and we communicate everything on each patient.” (10) “…we have intentionally made or planned a clinical team meeting on a weekly basis for each kind of program that includes anything from the people who are in contact, one-to-one, with the clients. [These meetings] include the interns training with us and the team lead, and so on… it’s not based on crisis or incident report. It’s really just part of the practice.” (24)
	Communicating as needed	“So making sure that we’re able to communicate that way about any client concerns and any updates… Anytime, anywhere, really….we’re all in person. We’re not virtual. So it’s very easy to just go and have a conversation with another staff person.” (5) “There are a lot of casual conversations. Sometimes, if I meet with a patient and I notice something in that conversation that’s a little off, I might find the nurse and say, ‘Hey, how is this person looking to you today? I notice they said this thing today, and it was concerning.’… A lot of the communication is just kind of casually checking in with each other on the patients.” (8)
**Understanding and identifying “teaming” in various ways**		
Varied perceptions of teaming		
	Who’s on the team	“The teamwork that we have not only within our [methadone] team but also with our family medicine team, is like one of the greatest strengths I think that we have” (3) “We are constantly working cooperatively with the police department, with the public school system, with the local faith-based organizations, with the Health District, with our Park and Rec department, the Y.M.C.A. And then accessing state and local resources treatment programs” (13)
	How teaming is understood	*Cognitive framework:* “Thinking in a teaming way” is teaming (2) *Ongoing dialogue:* “In 24-h level of care with the pressure that we have on ourselves, and you know, from insurance and everybody, to move them through treatment at kind of a rapid pace. We need to figure out meds *now*… When you’re moving [clients] through things in an accelerated way, you’re running into side effects and challenges…these constant shifts result in needing more communication, just like all the time, like, it's just always trying to keep up with all the constant shifts” (21) *Shared treatment goals:* “the goal is to keep us communicating so that we stay on the same page, and we’re supportive for the patients.” (4)

Trustworthiness was addressed in this study per the standards developed by Lincoln and Guba.^26^ The researchers developed a narrative statement that summarized the emergent categories, which was emailed to participants for member checking. Participants were asked to provide feedback on the alignment between the statement and their experiences. Following two emailed reminders, five participants responded, all of whom said they agreed with the statement. Of note, one respondent highlighted the importance of hierarchy and power differentials among professions on a team, specifically as it relates to decision-making. This feedback was incorporated into the final model. In addition, researchers, who were from diverse professional and clinical backgrounds, also engaged in analytic memoing throughout the coding process for the purposes of bracketing and ensuring credibility. The final process of theory development was iterative diagram development, which led to the final “prism” model. One researcher proposed the diagram from discussions of categories, and all three researchers met to evaluate the theoretical abstraction and whether it continued to portray the meaning of the initial data. This process occurred three times before the model diagram was finalized.

## Results

### Behavioral health providers’ perceptions of teaming: a prism model

**Figure 1:**
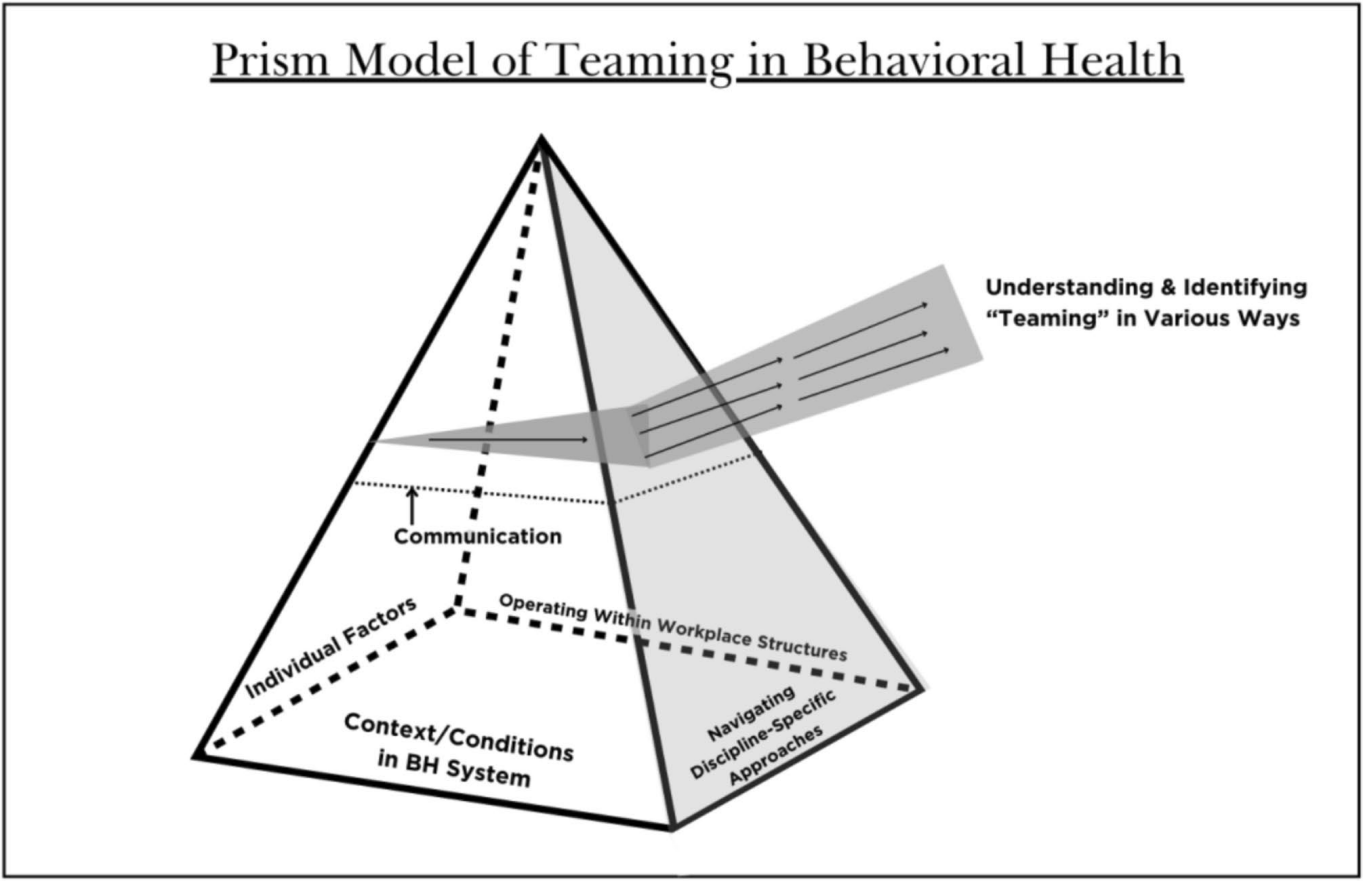
Prism model of teaming in behavioral health

Participants’ rich descriptions of their experiences working in various behavioral health settings led to the development of a prism model, which is three-dimensional and reflective that behavioral health providers exist within a complex and dynamic system. The prism model represents perceptions and experiences of factors related to teaming and illustrates the complex interplay of those factors (see Fig. 1). The prism captures both individual and systemic components of teaming, showing how teaming is understood by participants beyond a binary (of happening or not happening).

The specific model components: behavioral health context, individual factors, navigating discipline-specific approaches, workplace structures, communication as a “throughline,” and varied perceptions of teaming are further described below, with key quotes identified in Table 3. Transcript numbers are provided throughout the table and discussion.

### Behavioral health context

The larger context that underpins behavioral health care in the USA was identified by respondents as impacting how services are delivered, including opportunities for teaming. Themes included the siloed nature of care delivery and financial/reimbursement systems. As the quotes in Table 3 indicate, these themes are highly interconnected and also related to other categories such as workplace factors. While some participants described multiple programs and/or levels of care being present within the same organization (11, 18, 19, 20), they describe barriers to coordination between programs, such as “lack of time” (10) and “lack of resources” (5). Fragmentation and system barriers for continuity of care outside of agencies are even greater, with participants describing the lack of community-based resources such as outpatient providers and programs (9).

Participants also described difficulties with reimbursement for team-based care because insurance often does not cover collaborative approaches or team meetings (23). The fee-for-service structure also puts pressure on individual providers to meet productivity expectations that lead to burnout and turnover. “Scheduling also impacts, like the ability to collaborate in between clients, because you barely have the time to use the restroom” (20). Overall, participants indicated that the financial systems, cultural expectations, and models of care are often barriers to teaming.

### Individual factors

Many respondents indicated that individual clinicians’ experiences and expertise were more important than their professional disciplines in clinical work. In settings that included recovery specialists, their lived experiences were particularly valued (3, 9), and bilingual and bicultural clinicians enhanced clinical care through providing contextual, culturally sensitive care. At the same time, bilingual/bicultural clinicians also experienced additional burdens and the need to set boundaries, as they were asked to do things like interpretation, which was outside their scope of practice (16, 17).

Additionally, several participants shared about the ways in which effective teaming required navigating individual team members’ personalities and preferred work and communication styles (9, 12, 13). Personal characteristics such as “maturity” and skills in building relationships (e.g., being friendly as saying “hello” (6)) and having the perspective that “somebody always has something to offer that can be helpful” (13) were seen as making some people “better team players” (4), while “aggressive” personalities were seen as a barrier to teaming (13). For professions with more power in the medical hierarchy (e.g., psychiatrists), listening to other perspectives was seen as an individual choice (21) rather than a professional mandate.

### Navigating discipline-specific approaches

When examining the various roles of behavioral health clinicians in a teaming environment, there were several key factors. Participants were clear that there was a divide between those on the team with a physical health background and those without (1, 2, 3, 7). This was carried over into a divide between those clinicians who could prescribe medication and those who could not (5, 22).

This separation was not just a division but sometimes expressed as more of a hierarchy by participants. Several master’s-level clinicians felt that they did not have the “authority” to weigh in on medical concerns, especially since decisions about discharge and dispositions seemed to rest on psychiatrists, MDs, or APRNs (9). One participant stated that being able to communicate within a hierarchy comes from feeling you are valued (18). Certain participants who were not able to prescribe talked about wanting more knowledge of the medical side of treatment in order to be able to team (17), especially when it came to certain “styles of talking” (19). However, others seemed to indicate that it was on the prescriber to be “open” to working collaboratively with non-prescribing team members (21).

One of the main themes of the master’s-level clinicians (LCSWs, LPCs, and LMFTs) was that their roles were interchangeable on the team (2, 12, 23, 3, 20, 5, 13). Many reported having the same professional title, for example, “clinician” (2, 6, 20) or “therapist” (3, 15), and those differences stemmed from individual theoretical orientation, experience, or expertise (2, 6, 13, 15).

### Workplace structures

Workplace structure greatly impacted the ability to team, according to participants. One of the key issues was the use of space at a worksite. Many participants indicated the need for some in-person space for a variety of reasons, including relationship-building, the opportunity for informal conversations, and being able to provide warm hand-offs (2, 9, 12, 23, 5, 14, 3). Several even suggested that a “small hub space” was preferable due to improvement in the ability to connect.

In addition, participants felt that routine, interdisciplinary meetings established by leadership were important (5, 23, 11). There were differences as to who was invited to these meetings and who had the time to attend the meetings. Several participants indicated that non-licensed professionals (e.g., techs and aides) were not included in weekly teaming meetings and that this was an impediment to care (21, 6). Others indicated that prescribers did not attend or were not expected to attend these weekly meetings. And yet, these psychiatrists may be final decision-makers on disposition or treatment plans for the client (9).

Another barrier to prescribers attending meetings was the scheduling of appointments by the worksite. Several participants reported that prescribing APRNs were so busy and “overscheduled” (23, 5) that they did not have the time to attend meetings or even be available to discuss clients with others. This issue with scheduling also was evident in master’s-level providers. Many participants indicated that lack of time due to productivity concerns of the worksite not only limited time to team but also increased burnout (20, 5). This burnout resulted in high turnover at those sites, which one participant felt impacted teaming (20).

### Communication as a “throughline”

Communication permeated throughout all participants’ descriptions of their lived experiences with “teaming”. Participants unanimously discussed communication as an essential ingredient for effective teaming; some also discussed communication as important to nurturing a cohesive team (8, 12). They reported that the quality and means of communication (or lack thereof), impacted team dynamics and client/patient care (4, 6–9, 12). As one interviewee emphasized, “It is all about the communication” (4).

Participants consistently reported engaging in numerous means of written communication throughout their workdays, especially in light of use of EMRs and a shift towards telehealth and remote work. As one participant stated, “…and email after email after email…back and forth, several hundred a day,” (1). However, some participants also shared limitations of written communication. For example, “…when we spoke more in person it was more human. It was more of a connection” (18).

Scheduled meetings were also commonly reported by participants, although the frequency and structure varied by setting. For instance, participants working in hospital and in-patient settings reported routine meetings in which all clinicians reviewed treatment plans for all clients/patients (10, 21). Some participants working in outpatient settings reported less frequent scheduled meetings or meetings with limited attendees and/or limited time to address client needs (18–20). Other participants emphasized the importance of having a balance between scheduled meetings and the ability to communicate flexibly (e.g. through “casual conversation” (8)) and as needed with other team members, in support of client care. Some suggested that it was easier to have these “as needed” conversations in person versus remotely (5, 14, 20).

### Understanding and identifying “teaming” in varied ways

In addition to core clinical treatment teams, some participants described teams that were “nested,” expanding beyond a primary team to integrate multiple primary teams. Some participants also had expansive views of who was a team member, including anyone providing services to a client, regardless of the agency that they were working for (2, 16, 17). As one participant noted, in an acute care setting, multiple in-house and external professionals contribute to treatment planning (9). One participant noted that having lawyers and DV advocates at the same agency improved client care (17). Likewise, mental health providers hired by a municipality helped to create expansive teams that included people traditionally (e.g., therapists in private practices) and not traditionally involved in behavioral health work (e.g., police officers, town administrators).

Some participants also spoke of the value of the “lived experience” that peer recovery coaches bring to the team (3) and that roles such as peer counselors and care coordinators allow for “upward mobility” if included as team members (4). On the other hand, there were settings in which teaming was restricted to licensed clinicians, and paraprofessionals were not included in team meetings (21), and some participants indicated that they felt the need to further educate non-licensed colleagues about mental health issues in support of effective client care (8). While several participants spoke about the importance of client-centered care (3, 4, 7), emphasizing treatment approaches that are determined based on client needs rather than the professional providing care (13), clients were not explicitly identified as team members. In fact, sometimes clients were named as barriers to teaming because of symptomology (3, 8, 21).

Finally, participants shared varied perceptions of what teaming means in behavioral health settings. Although participants did not explicitly disagree with the definition of teaming provided by researchers, they spoke about their own conceptualizations of teaming, which were varied and often seemed to be influenced by agency culture and expectations. These included: cognitive conceptualization of teaming (2), teaming as an ethical imperative (2), teaming as a core value for effective care and sustainability for clinicians (24), teaming as an ongoing dialogue among colleagues where many perspectives can be voiced (4, 21), and teaming as working on shared treatment goals (4). One participant shared the importance of “professional humility” and being open to other viewpoints as necessary for teaming as dialogue (3). Other conceptualizations of teaming included: information sharing but not necessarily collaboration (19), consultation but not necessarily shared treatment planning (15, 18–20), and employee input and feedback to administration (6). Alternately, some participants described teaming as not feasible in practice (particularly private practices, 13) because team meetings and conversations are not billable (20). Collaborative practices might be written “on paper” but not implemented “in practice” (22).

## Discussion

The prism model that the researchers developed from the data about participants’ experiences with teaming in behavioral health settings is aligned with previous literature that highlights the multi-faceted nature of teaming in physical health and integrated primary care settings and the importance of context and culture.^15^ For example, participants highlighted the important influences of organizational culture as well as relationship-building in the unique experiences of teaming in a particular location. This aligns with findings from integrated primary care research.^19,21^ Unique findings of this research specific to behavioral health include participants’ identification of individual clinicians’ cultural identities and physical space as being key influencers of teaming.

Also unique is the prism model itself. It is dynamic, acknowledging the role of the individual in the system while also recognizing that unique participant perceptions of teaming are shaped by environment and contextual forces such as disciplinary approaches and workplace structures. Each pathway is singular, with a variety of interacting factors; there is the possibility for pathways to shift if various factors change. For example, one participant received internship training in team-based care, which led her to work professionally in a more integrated, inpatient environment. She spoke about the importance of having the skills to navigate various working styles of colleagues and the ability to operate within power dynamics historically present in medical settings. Her perception of teaming (shared treatment planning) was expansive, including community members, peer support, and co-workers. Another participant, who was bicultural and bilingual, highly valued her clinical work but had fewer opportunities to team in an outpatient setting, particularly because the community had limited programs/providers available to provide bilingual services. She viewed non-prescribing mental health professionals as interchangeable (as did most participants in the study). Her perception of teaming could be summarized as “consultation,” a term that is often used in psychological literature to denote the provision of information and guidance.^27^

These examples demonstrate a key finding. While teaming was viewed positively (many participants described wanting more professional collaboration and relationship-building), teaming was understood very differently by various participants, and in some settings, it seemed that little to no teaming was occurring. There is some literature that explores varied configurations and practices of teams in health settings, suggesting that they are better understood as “knotworks” rather than “networks,” given the more fluid practices and configurations occurring in current systems.^28^ However, as researchers and practitioners trained in a traditional integrated care model, the authors were initially “blind” to this variation, presenting an “academic” definition of teaming in the interview guide.^8^ In sitting with the data, researchers began to see the varied and unique ways in which participants were describing their experiences with teaming in practice. These varied from “not happening” to “conceptualizing practice in a teaming way, even when real-world opportunities for teaming were intermittent” to “communication between clinicians and administrators,” to “ongoing dialogue with colleagues in primary teams, community partners, and clients themselves.” Participants were more likely to describe perceptions of expansive teaming in inpatient settings or specialty treatment settings (e.g., eating disorder, methadone). To the authors’ knowledge, these varied ways of describing teaming are not all present in the current literature.

Of particular interest are participants’ teaming descriptions that indicated limited or non-existent teaming (e.g., a therapist sending a referral email to a psychiatrist who is so tightly scheduled that there is no time to speak by phone, or supervisors providing case information in monthly staff meetings that all clinicians are not invited to). The synergy of previous research, researchers’ experiences as researcher-clinicians, and participants’ comments about contextual factors suggest that the following constructs may be at play: the impact of the medical model, which values medical ways of knowing above “recovery” and psychosocial ways of knowing; the limitations of fee-for-service billing, which makes billing for team meetings or collaborative conversations largely impossible; the American value of individualism above collectivism in health care; and the sometimes misplaced faith in technology to improve functionality.^29,30^ Further investigation is warranted.

Despite feeling “overwhelmed” at times with email and clinical documentation expectations, the vast majority of participants highlighted the importance of communication in the workplace. While several participants acknowledged that Zoom and other technological platforms can overcome physical constraints on meetings, many people voiced preferences for “face-to-face” meetings. The value that participants placed on relationship-building, which they said was better accomplished in person in shared work environments, was striking. This finding also dovetails with prior research indicating that perceived interdependence among mental health providers of various disciplines is positively associated with workplace satisfaction, knowledge sharing, and collaboration.^31^ Further research is warranted about the impact of tele-behavioral health on collaborative care.

## Limitations

The findings of this study can be fully understood within the framework of some limitations. As in all qualitative research, the results are limited as to generalizability, even though trustworthiness measures were employed. Also, participants were from a limited geographical region, and although efforts were made to diversify the behavioral health professions represented, interviews were mostly conducted with social workers and counselors. Researchers chose to limit participation in the study to licensed behavioral health providers rather than non-licensed providers, who may have provided a different perspective. Finally, the three authors represent two behavioral health professions (social workers and counselors), and although they attended to biases throughout the study, researchers are aware that their unique professional perspectives may influence the results.

## Implications for Behavioral Health

Findings from this study have implications for behavioral health education, practice, and research. It was notable that despite positive views of teaming, the vast majority of participants had not received training on interprofessional collaboration in graduate school. Even the approximately one-third of participants who graduated in the past 5 years had largely been trained in silos, including in internship settings. There are many opportunities for graduate programs to better align with the realities of practice. Given societal aspirations for integrated care and the varied experiences of teaming that participants describe in the field, graduate programs must better prepare students for a variety of practice settings. Uni-professional and interprofessional coursework should provide opportunities to learn skilled and flexible communication, professional humility, and more information about the financial and cultural contexts of behavioral health and models of integration.^32^

Given that participants described non-prescribing, licensed behavioral health professionals as doing virtually indistinguishable work in clinical practice, reforms might include a “behavioral health professional” designation.^13^ Such a designation might clarify overlapping scopes of practice and reduce confusion for clients and community members.^14^ Additionally, the research found great variability in participants’ descriptions of capacity for teaming across settings. New models of behavioral health care that incentivize teaming, expand community supports and peer workforce, prioritize the goals of recovery and wellness, and provide opportunities for more flexible financing of collaborative behavioral health services are needed in the field.^33^ Some examples of promising models that incorporate these strategies include Certified Community Behavioral Health Centers (CCBHCs) and Innovations in Behavioral Health.^12,34^

Finally, there are many opportunities to advance research on teaming in behavioral health. These include the roles of individual and organizational culture in teaming, the impact of teaming on provider well-being, the impact of teaming on client outcomes, and further evaluation of new models of collaborative care.

This study offers a theory of factors impacting teaming in behavioral health, as identified by clinicians in behavioral health settings, a group that has been understudied in the integrated care and team literature. Unique descriptions of teaming point to clinicians’ efforts to engage in collaboration even in environments where it is not prioritized and there are limited opportunities. Understanding the dynamic factors that impact clinicians’ experiences of teaming can support educators, policymakers, and leaders in the field in identifying opportunities for impactful shifts toward more collaborative care.

## Data Availability

Data are not publicly available and cannot be shared due to participant confidentiality.
